# Cross-Neutralisation of Novel Bombali Virus by Ebola Virus Antibodies and Convalescent Plasma Using an Optimised Pseudotype-Based Neutralisation Assay

**DOI:** 10.3390/tropicalmed6030155

**Published:** 2021-08-25

**Authors:** Emma M. Bentley, Samuel Richardson, Mariliza Derveni, Pramila Rijal, Alain R. Townsend, Jonathan L. Heeney, Giada Mattiuzzo, Edward Wright

**Affiliations:** 1 Division of Virology, National Institute for Biological Standards and Control-MHRA, Blanche Lane, South Mimms EN6 3QG, UK; samuel.richardson@pirbright.ac.uk (S.R.); giada.mattiuzzo@nibsc.org (G.M.); 2 School of Life Sciences, University of Sussex, Brighton BN1 9QG, UK; m.derveni@hvivo.com (M.D.); ew323@sussex.ac.uk (E.W.); 3 MRC Human Immunology Unit, MRC Weatherall Institute of Molecular Medicine, Radcliffe Department of Medicine, University of Oxford, Oxford OX3 9DS, UK; pramila.rijal@rdm.ox.ac.uk (P.R.); alain.townsend@imm.ox.ac.uk (A.R.T.); 4 Department of Veterinary Medicine, University of Cambridge, Cambridge CB3 OES, UK; jlh66@cam.ac.uk

**Keywords:** ebolavirus, pseudotyped virus, cross-neutralisation

## Abstract

Ebolaviruses continue to pose a significant outbreak threat, and while Ebola virus (EBOV)-specific vaccines and antivirals have been licensed, efforts to develop candidates offering broad species cross-protection are continuing. The use of pseudotyped virus in place of live virus is recognised as an alternative, safer, high-throughput platform to evaluate anti-ebolavirus antibodies towards their development, yet it requires optimisation. Here, we have shown that the target cell line impacts neutralisation assay results and cannot be selected purely based on permissiveness. In expanding the platform to incorporate each of the ebolavirus species envelope glycoprotein, allowing a comprehensive assessment of cross-neutralisation, we found that the recently discovered Bombali virus has a point mutation in the receptor-binding domain which prevents entry into a hamster cell line and, importantly, shows that this virus can be cross-neutralised by EBOV antibodies and convalescent plasma.

## 1. Introduction

The 2013–2016 outbreak of Ebola virus in West Africa, which was declared a public health emergency of international concern (PHEIC) and caused over 28,000 cases and 11,000 deaths [1,2], re-focused the world’s attention on the public health threat the *Ebolavirus* genus represents. This included the establishment and inclusion of Ebola virus on the World Health Organization’s research and development blueprint [3], which prioritizes fundamental research and supports the development of vaccines and therapeutics along with preparedness activities to mitigate future outbreaks.

The first documented cases of viral haemorrhagic fever caused by an ebolavirus occurred in 1976, with nearly simultaneous outbreaks of Ebola virus (EBOV) [4] and Sudan virus (SUDV) [5] in Central Africa. Up until 2013, they had each caused sporadic outbreaks limited to this region [6,7] and this trend has continued since the PHEIC with a further 5 outbreaks of EBOV, as well as an outbreak in West Africa [8]. Human cases have also occurred with two further species identified; these are the Bundibugyo virus (BUDV) and the Taϊ Forest virus (TAFV). Distinctly, Reston virus (RESTV), which has been identified in primate and swine populations within the Philippines, is non-pathogenic in humans [9,10]. In 2018, as part of the PREDICT project to detect known and unknown viruses within wildlife reservoirs [11,12,13], the genome sequence of a further ebolavirus, Bombali virus (BOMV), was isolated from free-tailed bats in Sierra Leone [14]. Subsequent surveys have also found it to be present in bats in Kenya and Guinea [15,16,17]. Through *in situ* sequence analysis and the use of a recombinant vesicular stomatitis virus (rVSV) encoding the BOMV glycoprotein (GP), it was determined that entry into human cells occurs via the Niemann–Pick C1 (NPC1) receptor, similar to other ebolaviruses [14]. However, the potential to cause disease in humans remains unknown. 

Currently, EBOV-specific vaccines and antivirals have been licensed for use following expedited development [18]. Prior to this, supportive care and convalescent plasma had been applied as an empirical treatment during filovirus outbreaks [19,20]. Yet in light of the disease potential (i.e., documented human cases or ability to infect human cells *in vitro*) posed by all 6 *Ebolavirus* species, there are efforts to develop cross-protective candidates [21]. To evaluate their effectiveness and further study serological responses against existing and newly discovered ebolaviruses, species-specific neutralisation assays are required. The use of live ebolavirus is restricted to biosafety level 4 (BSL4) laboratories [22]; therefore, EBOV GP-pseudotyped virus (PV) has been used as an alternative, showing good correlation in the results between the live virus assay and the pseudotype system [23,24,25]. Previous work has suggested PV systems based on vesicular stomatitis virus (VSV) correlate better with the gold standard live neutralisation assay [26,27]. However, a caveat in these analyses is the variability of target cell lines used in the different reports. Here, we have optimised a lentiviral-vector based (LVV) neutralisation assay for EBOV antibodies and applied it in the evaluation of cross-neutralising antibodies against the other ebolavirus species, including BOMV.

## 2. Materials and Methods

### 2.1. Cell Lines and Virus Envelope Plasmids 

The highly transfectable human embryonic kidney 293T clone-17 cells (HEK 293T/17; American Type Culture Collection (ATCC), CRL-11268) were used for pseudotyped virus production as well as subsequent titration and neutralisation assays, together with a human lung carcinoma (A549; ATCC, CCL-185), African green monkey clone-E6 kidney (Vero E6; CRL-1586), feline kidney (CRFK; ATCC, CCL-94) and Chinese hamster ovary cell line (CHO-K1; ATCC, CCL-61). Cells were cultured in Dulbecco’s Modified Eagle Medium (DMEM) with high glucose and GlutaMAX (Gibco), except for CHO-K1 cells which were cultured in Ham’s F-12 nutrient mix (Gibco). Both were supplemented with 10% fetal calf serum (Pan-Biotech GmbH) and 1% penicillin/streptomycin (Sigma-Aldrich) and maintained at 37 °C, 5% CO_2_.

The vesicular stomatitis virus (VSV) envelope glycoprotein was cloned in the pMD2.G expression vector [28]. The envelope glycoprotein sequences of *Ebolavirus* species studied were each cloned within the pCAGGS expression vector [29]. This included the Ebola virus (EBOV) isolate Makona/GIN/2014/Kissidougou-C15 (GenBank accession: KJ660346), Bundibugyo virus (BDBV) isolate UGA/2007 (FJ217161) and Bombali virus (BOMV) isolates *Mops condylurus*/SLE/2016/PREDICT_SLAB000156 (MF319185), *Chaerephon pumilus*/SLE/2016/PREDICT_SLAB000047 (MF319186) and *Mops condylurus*/Kenya/B241 (MK340750), which were chemically synthesized (BioBasic, GeneWiz and GeneArt, respectively). The glycoprotein sequences of Tai Forest virus (TAFV) isolate CIV/1994 (FJ217162), Sudan virus (SUDV) isolate Boniface/SUD/1976 (FJ968794) and Reston virus (RESTV) isolate Pennsylvania/USA/1989 (AY769362) were kindly provided by Prof. Graham Simmons (Vitalant Research Institute). The S146P mutation was introduced into the BOMV envelope GP sequence (residue 142) via Q5 site-directed mutagenesis (New England Biolabs) and confirmed by Sanger sequencing. 

### 2.2. Human Plasma and Monoclonal Antibody Samples

The WHO 1st International Standard for Ebola virus antibodies–Sierra Leone Convalescent Plasma Pool (NIBSC catalogue code: 15/262) was reconstituted as per the instructions for use in 0.5 mL sterile distilled water to give a working potency of 1.5 IU/mL. Similarly, the WHO Reference Panel of anti-Ebola virus convalescent plasma (NIBSC catalogue code: 16/344) was reconstituted in 0.25 mL sterile distilled water. It comprises samples from four individuals donated through the American Red Cross (ARC; 15/280), UK National Health Service Blood and Transplant (NHSBT; 15/282), Oslo University, Norway (NOR; 15/284) and National Institute for Infectious Diseases Lazzaro Spallanzani, Italy (INMI, 15/286), as well as a negative control (NEG, 15/288). 

The broadly neutralising pan-ebolavirus mAb CA45 [30], isolated from an immunised cynomolgus macaque, was kindly provided by Dr. M. Javad Aman (Integrated BioTherapeutics). Two potently neutralising mAbs were derived from a vaccinated human [31], demonstrating some ebolavirus cross-reactivity, P6, or EBOV-specific, P7. 

### 2.3. Pseudotyped Virus Production and Titration

Pseudotyped virus production was based on methods previously described [28,32,33]. HEK 293T/17 cells were seeded into a 10 cm culture dish (Nunc) to reach 60–80% confluence after 24 h. The HIV-1 *gag-pol* plasmid, pCMV-Δ8.91, and the firefly luciferase reporter plasmid, pCSFLW, were then co-transfected with the pCAGGS plasmid-containing envelope sequence at a ratio of 1:1.5:1 μg, respectively, using 10.5 μL Fugene-HD transfection reagent (Promega) in 175 μL serum-free Opti-MEM (Gibco). Culture supernatant containing pseudotyped virus was harvested at 48 and 72 hrs post-transfection, then passed through a 0.45 μm pore filter (Millipore); aliquots were prepared for storage at −80 °C.

Titres of pseudotyped virus stocks were determined via end-point dilution on target cells. Starting from a 1:5 dilution, 5-fold serial dilutions were prepared within culture medium in triplicate across 8 points. Each dilution was transferred to a 96-well culture plate containing target cells seeded at 2 × 10^4^ cells/well and incubated for 60 hrs at 37 °C, 5% CO_2_. Following incubation, culture medium was removed and the target cells were incubated for 5 mins with a 1:1 mix of phenol-free DMEM (Gibco) and Bright-Glo reagent (Promega), with luciferase activity detected as relative light units (RLU) using a Glomax Navigator microplate luminometer (Promega). Titres were calculated as the 50% tissue culture infective dose (TCID_50_)/mL following the Spearman and Kärber method [34].

### 2.4. Neutralisation Assays

In a 96-well plate, doubling dilutions of human plasma or mAb sample, prepared in duplicate across 10 points from a 1:20 or 25 μg/mL starting dilution, respectively, were incubated with 50 TCID_50_ of pseudotyped virus for 1 hr at 37 °C and 5% CO_2_. Following incubation, the antibody and pseudotyped virus dilutions were added to target cells seeded in a 96-well culture plate at 2 × 10^4^ cells/well and incubated for a further 60 h at 37 °C and 5% CO_2_. Luciferase activity as RLU was detected as described above. Percentage neutralisation was calculated in GraphPad Prism, as described elsewhere [35], by normalising RLU data to pseudotyped virus and cell-only controls before non-linear regression analysis, fitting a 4-parameter dose-dependent inhibition curve to interpolate 50% inhibitory concentrations (IC_50_). 

### 2.5. Protein Structure Modelling and Sequence Alignment

Analysis of residues at the GP-NPC1 interface was performed using the crystal structure of EBOV GP bound to domain C of human NPC1 (PDB ID: 5F1B) [36], with 3D modelling and images generated in NGL viewer [37].

Sequence alignment used representative sequences of NPC1 from the species of target cell lines, including HEK293T/17—*Homo sapiens* (GenBank accession: AAB63982.1), CRFK—*Felis catus* (AAF72187.1) and CHO-K1—*Cricetulus griseus* (AAF31692.1). In addition to the ebolavirus GP sequences used experimentally, all publicly available *Bombali ebolavirus* sequences were identified from the NIAID Virus Pathogen Database and Analysis Resource (ViPR) [38] through the website at http://www.viprbrc.org/ (accessed on 15 July 2021) The NPC1 and ebolavirus GP sequences were each aligned using Clustal Omega [39] and then input into ESPript [40] to determine amino acid similarities of residues identified as involved in GP-NPC1 binding. 

## 3. Results

### 3.1. Target Cell Line Is Critical for Optimal Ebolavirus Pseudotype-Based Neutralisation Assay

**Figure 1:**
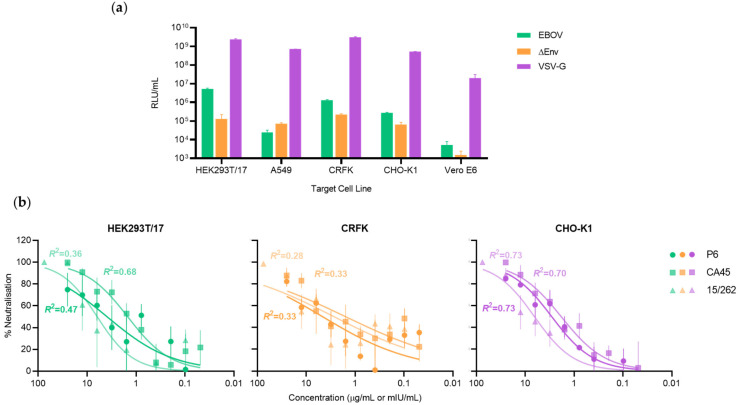
Comparison of EBOV-pseudotyped virus infectivity and neutralisation on various target cell lines. (**a**) Infectivity of the EBOV-pseudotyped lentiviral particles measured as relative luminescent units (RLU/mL) were assessed on 5 target cell lines. A vesicular stomatitis virus G protein (VSV-G) pseudotype was used as a positive control for the LVV system. LVV without an envelope protein (ΔEnv) was used to determine the background signal. (**b**) The percentage neutralisation of dilutions of monoclonal antibodies P6 or CA45 (μg/mL) and WHO IS (20/262; mIU/mL) following incubation with 50 TCID_50_ of EBOV-pseudotyped virus on each of the target cells, with the fit (*R*^2^) of the dose-response curve annotated. Error bars indicate SEM (*n* = 2).

Ebola virus lentiviral-pseudotyped virus (EBOV-LVV) incorporating a firefly luciferase reporter gene was titrated on different cell lines to select for those most permissive to EBOV-LVV entry (Figure 1a). EBOV-susceptible human HEK 293T/17 and A549 cells were evaluated in parallel to feline CRFK, hamster CHO-K1 and African green monkey Vero E6 cells. The EBOV-LVV reporter signal (RLU/mL) on Vero E6 was too low to be usable; this is not surprising, as monkey cells possess a restriction factor inhibiting post-entry steps of HIV-1-based particles [41]. Indeed, the VSV-G LVV control had a signal over 100-fold lower on Vero E6 than on the other cell lines. It is more unclear why the EBOV-LVV entry signal on human A549 was lower than the envelope-less LVV control.

Therefore, the 3 remaining cells lines, HEK 293T/17, CRFK and CHO-K1, were then used to evaluate EBOV-LVV neutralisation performance using a normalized input of 50 TCID_50_, with well-characterised anti-EBOV mAbs (P6 and CA45) and the WHO International Standard (IS) comprising a pool of convalescent plasma from Ebola-recovered individuals. Both visual and mathematical analysis of the fit of dose-dependent inhibition curves showed that performance was poor across all samples on CRFK cells, with an *R*^2^ value of 0.33 for each of the mAbs P6 and CA45 and 0.28 for the WHO IS plasma sample (Figure 1b). There was a similarly poor fit for the WHO IS plasma sample and the mAb P6 on HEK 293T/17 cells (*R*^2^ = 0.36 and 0.47, respectively). A superior dose-response profile was observed using the CHO-K1 target cell line, with a robust fit (*R*^2^ ≥ 0.70) across all samples tested, supporting its selection as the optimal target cell line to evaluate cross-reactivity across the ebolavirus-pseudotyped viruses.

### 3.2. CHO-K1 Cells Are Refractory to Bombali Virus Entry

**Figure 2:**
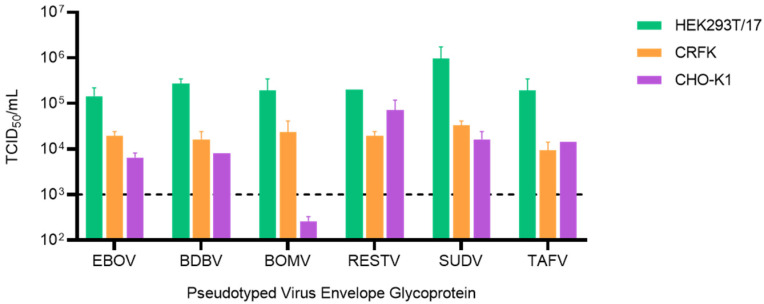
Comparison of ebolavirus-pseudotyped virus infectivity on different target cell lines. Titres calculated as TCID_50_/mL for pseudotyped virus incorporating ebolavirus envelope glycoproteins titrated on HEK293T/17, CRFK and CHO-K1 target cells. The dotted line indicates the minimum titre required for downstream neutralisation assays.

Following identification of the hamster cell line CHO-K1 as the optimal cell line for EBOV LVV-pseudotype neutralisation, we expanded the analysis of the neutralisation activity to other species of the genus Ebolavirus, including the most recently identified BOMV. Pseudotyped LVV titres were evaluated via end-point titration to determine TCID_50_/mL values on the HEK293T/17, CRFK and CHO-K1 target cell lines used for the EBOV-LVV neutralisation assay (Figure 2). All the Ebolavirus LVV presented the same pattern with the highest titres (>1 × 10^5^ TCID_50_/mL) obtained using HEK293T/17 cells. The titres on the CHO-K1 cells, previously identified as the most suitable target cells line for the neutralisation assay, were lower (3–60 fold), similar to titres on CRFK (8–30 fold reduction), but still suitable for the downstream neutralisation assays. The only exception was for the BOMV-LVV, which showed a 750-fold reduction when titrated on CHO-K1 cells, with a negligible titre of 259 TCID_50_/mL, which is not sufficient for performance of downstream neutralisation assays. 

### 3.3. Identification of a Critical Residue Involved in Bombali Virus Binding to the NPC1 Receptor

**Figure 3:**
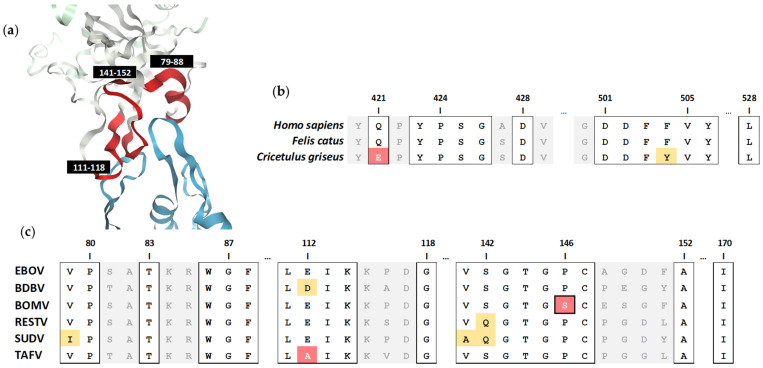
Analysis of NPC1 receptor binding to the ebolavirus glycoprotein. (**a**) The structure of human NPC1 receptor (blue) in complex with the EBOV glycoprotein (white) which highlights three NPC1-interacting regions (red) covering glycoprotein amino acids 79–88, 111–118 and 141–152. Alignment of the interface of (**b**) NPC1 sequence from HEK293T/17 (*Homo sapiens*), CRFK (*Felis catus*) and CHO-K1 (*Cricetulus griseus*) cell lines and (**c**) ebolavirus glycoprotein sequences, with residues in direct contact highlighted in squared boxes. Virus-specific polymorphisms are shaded yellow and red for amino acids similar or dissimilar to the consensus, respectively. The BOMV glycoprotein proline (P) to serine (S) polymorphism (P146S) is outlined.

To investigate the cause of CHO-K1 cells being refractory to BOMV entry, we looked to analyse sequence residues at the interface of ebolavirus GP binding to the NPC1 receptor (Figure 3a). Analysis of NPC1 residues in direct contact with the EBOV GP [36] from the different target cell species revealed mutations of two residues (Q421E and F504Y) within the *Cricetulus griseus* CHO-K1 cell line, which are conserved between the *Homo sapiens* HEK293T/17 and *Felis catus* CRFK cell lines (Figure 3b). While this may contribute to species-specific susceptibility to BOMV, sequence alignment of the interacting regions across the ebolavirus GP was undertaken to identify a determining factor (Figure 3c). The receptor-binding residues are highly conserved across the ebolaviruses, with a single proline to serine mutation (P146S) unique to the BOMV GP identified. Further analysis of the alignment of the five complete *Bombali ebolavirus* isolate sequences available reveals this mutation is common across four identified in the bat species *Mops condylurus*, yet the conserved proline occurs in a single isolate identified in another bat species, *Chaerephon pumilus* (Figure S1). Of note, attempts in our lab to produce pseudotyped virus expressing the BOMV GP isolated from the *C. pumilus* bat has not yielded titres high enough for downstream studies, while BOMV GP from both viruses isolated from *M. condylurus* achieved usable titres (Figure S2).

**Figure 4:**
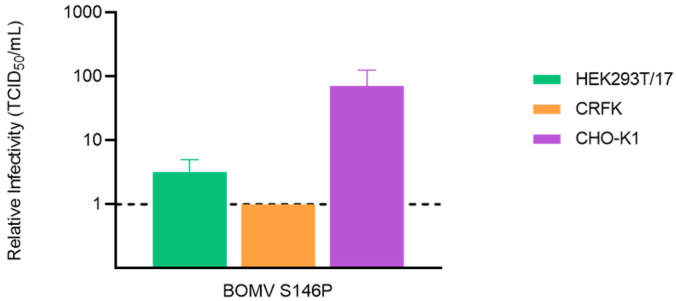
Restored infectivity of BOMV lentiviral-pseudotyped virus into CHO-K1 target cells after reversion of a serine at position 146 of the glycoprotein to a conserved proline (S146P). Infectivity, measured as TCID_50_/mL, calculated relative to a pseudotyped virus incorporating the wild type BOMV glycoprotein sequence on HEK293T/17, CRFK and CHO-K1 target cell lines. Error bars represent SEM (*n* = 2).

To test our theory, we performed site-directed mutagenesis to revert the serine at position 146 of the BOMV GP to the conserved proline. Pseudotyped virus was then produced with the mutant S146P alongside the wild type BOMV GP sequence and titrated on each of the target cell lines. As expected, the S146P mutation restored entry of BOMV pseudotyped virus into the CHO-K1 cell line, with no impact on entry into the alternative target cells (Figure 4). The titre on CHO-K1 cells was 70-fold higher, at 1.6 × 10^4^ TCID_50_/mL, than that of PV bearing the wildtype glycoprotein and was comparable to the titre of other ebolavirues on this target cell line (Figure 2). This confirmed the S146 residue is critical in the ability of BOMV to bind the NPC1 receptor of *C. griseus* and allowed entry to be restored for the performance of neutralisation experiments using the optimal CHO-K1 target cell line. 

### 3.4. Ebola Virus Convalescent Serum and Monoclonal Antibodies Cross-Neutralise Bombali Virus

**Figure 5:**
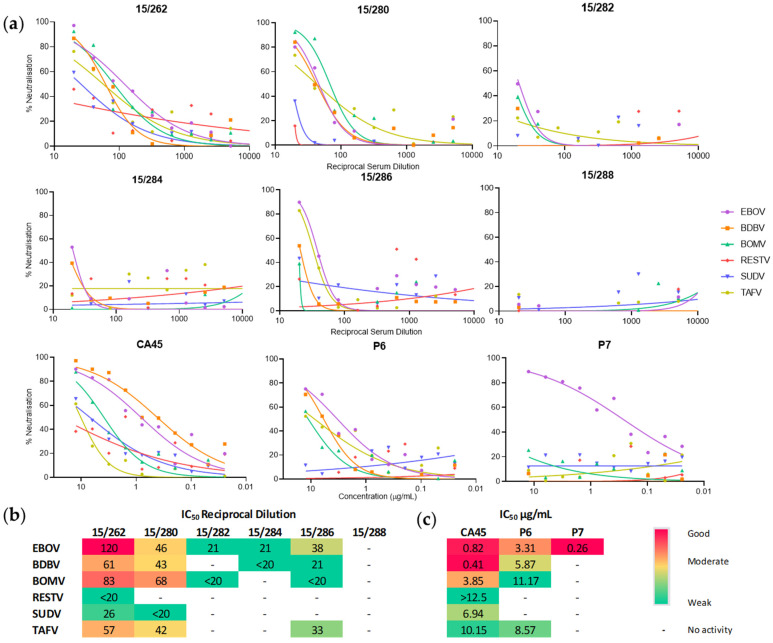
Cross-neutralisation of BOMV afforded by a panel of human EBOV convalescent serum and monoclonal antibodies. (**a**) Dose-response curves plotted as percentage neutralisation for dilutions of the WHO IS (15/262) and WHO Reference Panel members from four individuals recovered from EBOV (15/280, 15/282, 15/284, 15/286) and a negative control (15/288) along with two pan-ebolavirus mAbs (CA45, P6) and an EBOV-specific mAb negative control (P7). (*n* = 2) (**b**,**c**) 50% inhibitory concentrations (IC_50_) expressed as reciprocal of the dilution factor for plasma samples (**b**) or in μg/mL for mAb (**c**); IC_50_ were interpolated via non-linear regression analysis, with potency ranked good to weak based on a 50th percentile mid-point. Where the IC_50_ is outside the dilution range tested, values are reported as greater or less than the minimum serum (1:20) (**b**) and mAb (12.5 μg/mL) (**c**) dilution tested.

The level of cross-neutralisation afforded against BOMV in comparison to other ebolaviruses was evaluated using both convalescent plasma and existing cross-reactive mAbs. Given CHO-K1 are refractory to BOMV, BOMV P146 was used. The panel of convalescent plasma samples collected from survivors during the 2013-2016 EBOV outbreak in West Africa included a pool of high titre samples from patients in Sierra Leone (15/262) and individual samples of mid-low titres from repatriated patients (15/280–15/286), along with a negative control (15/288) [42]. Dose-response curves plotting the level of neutralisation across the dilutions tested shows a reducing degree of cross-reactivity based on titre of the sample tested (high-low), with four out of five samples cross-reacting with BOMV (Figure 5a). The high titre pool 15/262 cross-reacts with all the ebolavirus PV, with the BOMV IC_50_ of 83 being the second most potently neutralised in comparison to EBOV (IC_50_ 120), the virus of exposure (Figure 5b). Interestingly, in the case of the mid-titre sample 15/280, BOMV is the most potently neutralised PV, with an IC_50_ titre 1.5× higher than EBOV. 

Of the mAb samples tested, CA45 has been shown to have neutralising activity against EBOV, BDBV, RESTV and SUDV. This is supported by our data, with CA45 found to additionally have activity against BOMV and TAFV (Figure 5a), with its potency against BOMV ranked moderately (IC_50_ 3.85 μg/mL) amongst the panel of ebolavirus PV (Figure 5c). A study to isolate P6 from a human vaccinee showed that it had cross-reactivity against BDBV but not SUDV [31]; this again is supported by our data and extended to include both BOMV and TAFV (Figure 5a,c). In the same study, P7 was found to be EBOV specific and is used here as a control, with reactivity solely detected against the EBOV PV. In all cases except with the low potency sample 15/284, BOMV was found to be neutralised at a similar or more potent level than other ebolaviruses, which suggests likely efficacy of both existing and cross-reactive treatments under development. 

## 4. Discussion

Current ebolavirus vaccine and therapeutic development efforts are focused on cross-neutralising candidates [43,44]. Neutralising antibodies against the envelope GP are considered both a determinant of infection control and a possible correlate of protection [45,46,47]. To measure the potency of neutralising antibodies, the gold standard method is a live Ebola virus neutralisation assay, requiring BSL4 facilities and specialised operators. Implementation of pseudotyped virus, which has been shown to offer good levels of correlation with live virus assays [23,24,25], provides a suitable and safer alternative to investigate neutralisation activity of serological samples. An additional advantage is that their production only requires the envelope GP sequence of the virus of interest. That said, it is important to take steps to optimise pseudotyped virus production and downstream assays to ensure reliability of results [48,49].

It is well-understood that the choice of target cell line influences the titre of the pseudotyped virus stock, due to differences such as the cell surface receptor density or availability of required protease. Ebolaviruses are known to have a broad host range, with the Niemann–Pick C1 (NPC1) entry receptor being mostly ubiquitously expressed across non-lymphoid cells [50,51,52,53]. However, there is variability in the activity of cathepsin B and L proteases required for GP priming [54,55]. This likely contributes towards the differences in entry efficiency observed within this and other studies [26,56,57,58]. While African green monkey Vero E6 cells are commonly used for the propagation and assaying of live ebolavirus, they are refractory to LVV-based pseudotypes due to the presence of the intrinsic restriction factor, TRIM5ɑ, inhibiting HIV-1 post-entry steps [41]. This presents a caveat to studies reporting that pseudotypes based on a VSV core system, using Vero E6 target cells, offer better correlation with live ebolavirus neutralisation than that achieved with LVV pseudotypes on human HEK 293T/17 cells [26,27]. Data presented here showed that the hamster cell line CHO-K1, despite sustaining lower EBOV-LVV infectivity titres than the most commonly used HEK 293T/17, allowed for a more consistent neutralisation assay producing better fit dose-response curves both for monoclonal antibodies and convalescent plasma. 

The use of CHO-K1 as target cells for other species of ebolaviruses revealed that the recently discovered BOMV was the only pseudotype with impaired entry. Further investigation highlighted that one mutated residue in the BOMV GP S142 (P146 in the EBOV sequence) is primarily responsible for the inability of BOMV-pseudotyped virus to infect hamster cell lines. It is possible that the mutation of this proline residue impacts binding by causing structural changes to the receptor-binding domain, as is proposed to be the case for other ebolavirus polymorphisms, including a proline at nearby position 148 [59]. Other studies also show that single amino acid changes within the EBOV GP [60] and NPC1 receptor [61,62] can have marked effects on species-specific virus tropism. Of note, S142 is not present in the single BOMV sequence isolated from *C. pumilus*. It has not been possible to produce viable pseudotyped virus with this envelope, which may be attributed to mutations within the highly conserved coiled-coil domain shown to impact GP binding [63] (Figure S1); this warrants further analysis. Yet, it is conceivable that S142 may represent an adaptation of BOMV to *M. condylurus* NPC1. It has previously been suggested that each ebolavirus is adapted to a specific bat host [62], with variation occurring across the NPC1 binding domain, although a partial *M. condylurus* NPC1 sequence shows F504 is conserved [64]. 

To include the BOMV-pseudotyped virus in our analysis of cross-neutralising activity of EBOV antibodies and convalescent plasma, the S146 in the BOMV GP was mutated to a proline. We tested three monoclonal antibodies, two with known cross-ebolavirus species neutralising activity which target the fusion loop (CA45) and glycan cap (P6) and one which is EBOV-specific and binds the receptor binding domain (P7), and a panel of convalescent plasma from Ebola virus disease survivors collected following the EBOV outbreak in 2013-2016. For the monoclonal antibodies, our results confirm what has been previously reported in the literature for CA45 [30] and P6 [31] and expands their efficacies to BOMV and TAFV. Interestingly, BOMV was also neutralised by most of the EBOV convalescent plasma samples. This builds upon studies that have shown a pan-ebolavirus neutralising response can be mounted from natural infection [65] and existing therapeutic mAb candidates can neutralise BOMV [66], supporting the feasibility of developing cross-protective vaccines and therapeutics able to target all species within the ebolavirus genus.

## Data Availability

The data presented in this study are available on request from the corresponding author.

## Supplementary Materials

The following are available online at https://www.mdpi.com/article/10.3390/tropicalmed6030155/s1: Supplementary Figure S1. Sequence alignment of the EBOV GP sequence to four BOMV isolates GP; Supplementary Figure S2. Mean titres of lentiviral pseudotypes bearing different Bombali ebolavirus (BOMV) isolate GP.
