# Supplementary material for: Cross-Neutralisation of Novel Bombali Virus by Ebola Virus Antibodies and Convalescent Plasma Using an Optimised Pseudotype-Based Neutralisation Assay
Source: Trop Med Infect Dis. 2021 Aug 25;6(3):155. doi: 10.3390/tropicalmed6030155 (PMC8412100; doi:10.3390/tropicalmed6030155)
Supplement: Supplementary file 1 [file tropicalmed-06-00155-s001.zip › tropicalmed-1342011-supplementary.pdf]

```
EBOV/Makona/GIN/2014/Kissidougou-C15           MGVTGILQLPRDRFKRTSFFLWVIILFQRTFSIPLGVIHNSTLQVSDVDKLVCRDKLSST  60
BOMV/M.condylurus/SLE/2016/PREDICT_SLAB000156  ----MILQVPEKRHQRTVLFIWLVILFQRAVSVPLGVIHNSTLQVSDIDKFVCHDKLTST  56
BOMV/C.pumilus/SLE/2016/PREDICT_SLAB000047     ----MILQVPEKRHQRTVLFIWLVILFQRAVSVPLGVIHNSTLQVSDIDKLVCHDKLTST  56
BOMV/M.condylurus/Kenya/2018/B241              ----MILQVPEKRHQRTVLFIWLVILFQRAVSVPLGVIHNSTLQVSDIDKLVCHDKLTST  56
BOMV/M.condylurus/Kenya/2019/X030              ----MILQVPEKRHQRTVLFIWLVILFQRAVSVPLGVIHNSTLQVSDIDKLVCHDKLTST  56
BOMV/M.condylurus/Kenya/2019/Z153              ----MILQVPEKRHQRTVLFIWLVILFQRAVSVPLGVIHNSTLQVSDIDKLVCHDKLTST  56
                                                    ***:*..*.:** :*:*::*****:.*:*************:**:**:***:**

EBOV/Makona/GIN/2014/Kissidougou-C15           NQLRSVGLNLEGNGVATDVPSATKRWGFRSGVPPKVVNYEAGEWAENCYNLEIKKPDGSE  120
BOMV/M.condylurus/SLE/2016/PREDICT_SLAB000156  NQLRSIGLNLEGNGIATDVPSATKRWGFRAGVPPKVVGYEAGEWAENCYNLEIKKPDGSE  116
BOMV/C.pumilus/SLE/2016/PREDICT_SLAB000047     NQLRSIGLNLEGNGIATDVPSATKRWGFRAGVPPKVVGYEAGEWAENCYNLEIKKPDGSE  116
BOMV/M.condylurus/Kenya/2018/B241              NQLRSIGLNLEGNGIATDVPSATKRWGFRAGVPPKVVGYEAGEWAENCYNLEIKKPDGSE  116
BOMV/M.condylurus/Kenya/2019/X030              NQLRSIGLNLEGNGIATDVPSATKRWGFRAGVPPKVVGYEAGEWAENCYNLEIKKPDGSE  116
BOMV/M.condylurus/Kenya/2019/Z153              NQLRSIGLNLEGNGIATDVPSATKRWGFRAGVPPKVVGYEAGEWAENCYNLEIKKPDGSE  116
                                               *****:********:*************:*******.********************

EBOV/Makona/GIN/2014/Kissidougou-C15           CLPAAPDGIRGFPRCRYVHKVSGTGPCAGDFAFHKEGAFFLYDRLASTVIYRGTTFAEGV  180
BOMV/M.condylurus/SLE/2016/PREDICT_SLAB000156  CLPMAPEGIRGFPRCRYVHKVSGTGSCESGFAFHKEGAFFLYDRLASTIIYRGTTFAEGV  176
BOMV/C.pumilus/SLE/2016/PREDICT_SLAB000047     CLPMAPEGIRGFPRCRYVHKVSGTGPCESGFAFHKEGAFFLYDRLASTIIYRGTTFAEGV  176
BOMV/M.condylurus/Kenya/2018/B241              CLPMAPEGIRGFPRCRYVHKVSGTGSCESGFAFHKEGAFFLYDRLASTIIYRGTTFAEGV  176
BOMV/M.condylurus/Kenya/2019/X030              CLPMAPEGIRGFPRCRYVHKVSGTGSCESGFAFHKEGAFFLYDRLASTIIYRGTTFAEGV  176
BOMV/M.condylurus/Kenya/2019/Z153              CLPMAPEGIRGFPRCRYVHKVSGTGSCESGFAFHKEGAFFLYDRLASTIIYRGTTFAEGV  176
                                               *** **:****************** *  ..****************:**********

EBOV/Makona/GIN/2014/Kissidougou-C15           VAFLILPQAKKDFFSSHPLREPVNATEDPSSGYYSTTIRYQATGFGTNETEYLFEVDNLT  240
BOMV/M.condylurus/SLE/2016/PREDICT_SLAB000156  VAFIILPKAEKNFLQPPLTQGPTNTTNDPSSMYHSTTLEYETTRFGTNRSAFWFKVDNLT  236
BOMV/C.pumilus/SLE/2016/PREDICT_SLAB000047     VAFIILPKAEKNFLQPPLTQGPTNTTNDPSSMYHSTTLEYETTRFGTNRSAFWFKVDNLT  236
BOMV/M.condylurus/Kenya/2018/B241              VAFIILPKAEKNFLQPPLTQGPTNTTNDPSSMYHSTTLEYETTRFGTNRSAFWFKVDNLT  236
BOMV/M.condylurus/Kenya/2019/X030              VAFIILPKAEKNFLQPPLTQGPTNTTNDPSSMYHSTTLEYETTRFGTNRSAFWFKVDNLT  236
BOMV/M.condylurus/Kenya/2019/Z153              VAFIILPKAEKNFLQPPLTQEPTNTTNDPSSMYHSTTLEYETTRFGTNRSAFWFKVDNLT  236
                                               ***:***:*:*:*:.    : *.*:*:**** *:***:.*::* ****.: : *:*****

EBOV/Makona/GIN/2014/Kissidougou-C15           YVQLESRFTPQFLLQLNETIYASGKRSNTTGKLIWKVNPEIDTTIGEWAFWETKKNLTRK  300
BOMV/M.condylurus/SLE/2016/PREDICT_SLAB000156  FVQLESRFTPQFLVELNETIYIEGKRSNTTGRLIWQVNSRVDTDVGEWAFWENKKNLKKS  296
BOMV/C.pumilus/SLE/2016/PREDICT_SLAB000047     FVQLESRFTPQFLVELNETIYIEGKRSNTTGRLIWQVNSRVDTDVGEWAFWENKKNLKKS  296
BOMV/M.condylurus/Kenya/2018/B241              FVQLESRFTPQFLVELNETIYIEGKRSNTTGRLIWQVNSRVDTDVGEWAFWENKKNLKKS  296
BOMV/M.condylurus/Kenya/2019/X030              FVQLESRFTPQFLVELNETIYIEGKRSNTTGRLIWQVNSRVDTDVGEWAFWENKKNLKKS  296
BOMV/M.condylurus/Kenya/2019/Z153              FVQLESRFTPQFLVELNETIYIEGKRSNTTGRLIWQVNSRVDTDVGEWAFWENKKNLKKS  296
                                               :***********::****** .********:***:** .:** :*******.****.:.

EBOV/Makona/GIN/2014/Kissidougou-C15           IRSEELSFTAVSNGPKNISGQSPARTSSDPETNTTNEDHKIMASENSSAMVQVHSQGRKA  360
BOMV/M.condylurus/SLE/2016/PREDICT_SLAB000156  FPREELSLTAVPRAADSEHDAHPPEYTPGPDSNPTINDNTELVTEDPAHLVQLQRQGRKE  356
BOMV/C.pumilus/SLE/2016/PREDICT_SLAB000047     FPREELSLTAVPRAADSEHDAHPPEHTPGPDSNPTINDNTELVTEDPAHLVQLQRQGRKE  356
BOMV/M.condylurus/Kenya/2018/B241              FPREELSLAAVPRAADSEHDTHPPEHTPGPDSNPTINDNTELVTEDPAHLVQLQRQGRKE  356
BOMV/M.condylurus/Kenya/2019/X030              FPREELSLAAVPRAADSEHDTHPPEHTPGPDSNPTINDNTELVTEDPAHLVQLQRQGRKE  356
BOMV/M.condylurus/Kenya/2019/Z153              FPREELSLAAAPRAADSEHDAHPPKHTPGPDSNPTINDNTELVTEDPAHMVQLQRQGRKE  356
                                               :  ****::*. .. ..  .  * . : .*::* * :*:. :.:*: : :**:: ****

EBOV/Makona/GIN/2014/Kissidougou-C15           AVSHLTTLATISTSPQPPT-TKTGPDNSTHNTPVYKLDISEATQVGQHHRRADNDSTASD  419
BOMV/M.condylurus/SLE/2016/PREDICT_SLAB000156  ILPTTIPQAIE---REPPAAQHDNPRNSPTPPSPIESDITDSTQAEDLTHTDDPSTINSA  413
BOMV/C.pumilus/SLE/2016/PREDICT_SLAB000047     ILPTPTPQAIE---REPPAAQHDNPRNSPTPPSPIESDITGSTQAEDLTHTDDPSTINSA  413
BOMV/M.condylurus/Kenya/2018/B241              ILPTPIPQAIE---REPPAAQHDNPRNSPTPPSPIESDITDSTQAEDLTHADDPSTINSA  413
BOMV/M.condylurus/Kenya/2019/X030              ILPTPIPQAIE---REPPAAQHDNPRNSPTPPSPIESDITDSTQAEDLTHADDPSTINSA  413
BOMV/M.condylurus/Kenya/2019/Z153              ILPTPIPQAIE---REPPAAQHDNPRNSPTPPSPTESDITDSTQAEDLTHADDPSTINSA  413
                                                :      *      :**:  : .* **       : **: :**. :  :  * .:  *

EBOV/Makona/GIN/2014/Kissidougou-C15           TPPATTAAGPLKAENTNTSKSADSLDLATTTSPQNYSETAGNNN----THHQD--TGEES  473
BOMV/M.condylurus/SLE/2016/PREDICT_SLAB000156  TEEPLPEVGETTQ----VPRDPEGPRRTQPTPPTGQPEQPSDNTMTPGQIHSESAAPMGE  469
BOMV/C.pumilus/SLE/2016/PREDICT_SLAB000047     TEKPLPEVGGTTQ----VPRDPEDPRRTQPTPPTGQPEQPSDNTMTPGQIHSESAAPMGE  469
BOMV/M.condylurus/Kenya/2018/B241              TEESLPEVGGITQ----VPRDPEDPRITLSTPPTSQPEQPSDNTMTPGQIHSESAAPMGE  469
BOMV/M.condylurus/Kenya/2019/X030              TEESLPEVGGITQ----VPRDPEDPRITLSTPPTSQPEQPSDNTMTPGQIHSESAAPMGE  469
BOMV/M.condylurus/Kenya/2019/Z153              TEEPLPEVGGITQ----VPRDPEDPGITLPTPPTSQPEQPSDNTMTPGQIHSESAAPMGE  469
                                               *      .*  .      . :. :.   :  * * .  *  .:*.      *.:  :   .

EBOV/Makona/GIN/2014/Kissidougou-C15           ASSGKLGLITNTIAGVAGLITGGRRTRREVIVNAQPKCNPNLHYWTTQDEGAAIGLAWIP  533
BOMV/M.condylurus/SLE/2016/PREDICT_SLAB000156  RSIDGPGLLTNTLAGVARLITGAGRAKRESPEIRGAKCNPNLHYWTTHEESAAAGLAWIP  529
BOMV/C.pumilus/SLE/2016/PREDICT_SLAB000047     RSIDGPGLLTNTLTGVARLITGAGRAKRESPEIRGAKCNPNLHYWTTHEESAAAGLAWIP  529
BOMV/M.condylurus/Kenya/2018/B241              RSIDGPGLLTNTLAGVARLITGAGRAKRESPEIRGAKCNPNLHYWTTHEESAAAGLAWIP  529
BOMV/M.condylurus/Kenya/2019/X030              RSIDGPGLLTNTLAGVARLITGAGRAKRESPEIRGAKCNPNLHYWTTHEESAAAGLAWIP  529
BOMV/M.condylurus/Kenya/2019/Z153              RSIDGPGLLTNTLAGVARLITGAGRAKRESPEIRGAKCNPNLHYWTTHEESAAAGLAWIP  529
                                                *  .  **:***::*** ****. *::**       ***********::*.** ******

EBOV/Makona/GIN/2014/Kissidougou-C15           YFGPAAEGIYTEGLMHNQDGLICGLRQLANETTQALQLFLRATTELRTFSILNRKAIDFL  593
BOMV/M.condylurus/SLE/2016/PREDICT_SLAB000156  YFGPAAEGIYTEGLMQNQNELICGLRQLANETTQALQLFLRSTTELRTFSILNRKAIDFL  589
BOMV/C.pumilus/SLE/2016/PREDICT_SLAB000047     YFGPAAEGIYTEGLMQNQNELICGPRQLANETTQALQLFLRSTTELRTFSILNRKAIDFL  589
BOMV/M.condylurus/Kenya/2018/B241              YFGPAAEGIYTEGLMQNQNELICGLRQLANETTQALQLFLRSTTELRTFSILNRKAIDFL  589
BOMV/M.condylurus/Kenya/2019/X030              YFGPAAEGIYTEGLMQNQNELICGLRQLANETTQALQLFLRSTTELRTFSILNRKAIDFL  589
BOMV/M.condylurus/Kenya/2019/Z153              YFGPAAEGIYTEGLMQNQNELICGLRQLANETTQALQLFLRSTTELRTFSILNRKAIDFL  589
                                               **************:**: **** ***************:*****************

EBOV/Makona/GIN/2014/Kissidougou-C15           LQRWGGTCHILGPDCCIEPHDWTKNITDKIDQIIHDFVDKTLPDQGDNDNWWTGWRQWIP  653
BOMV/M.condylurus/SLE/2016/PREDICT_SLAB000156  LQRWGGTCRILGPDCCIEPHDWTKNITDRIDQIIHDFVDKPLPDQSNNDNWWTGWRQWIP  649
BOMV/C.pumilus/SLE/2016/PREDICT_SLAB000047     LQRWGGTCRILGPDCCIDPHDWTKNITDRIDQIIHDFVDKPLPDQSNNDNWWTGWRQWIP  649
BOMV/M.condylurus/Kenya/2018/B241              LQRWGGTCRILGPDCCIEPHDWTKNITDRIDQIIHDFVDKPLPDQSNNDNWWTGWRQWIP  649
BOMV/M.condylurus/Kenya/2019/X030              LQRWGGTCRILGPDCCIEPHDWTKNITDRIDQIIHDFVDKPLPDQSNNDNWWTGWRQWIP  649
BOMV/M.condylurus/Kenya/2019/Z153              LQRWGGTCRILGPDCCIEPHDWTKNITDRIDQIIHDFVDKPLPDQSNNDNWWTGWRQWIP  649
                                               ********:********:**********:*********** ****.:************

EBOV/Makona/GIN/2014/Kissidougou-C15           AGIGVTGVIIAVIALFCICKFVF                                       676
BOMV/M.condylurus/SLE/2016/PREDICT_SLAB000156  AGIGVVGVIAAFIALICICKIIC                                       672
BOMV/C.pumilus/SLE/2016/PREDICT_SLAB000047     AGIGVVGVIAAFIALICICKIIC                                       672
BOMV/M.condylurus/Kenya/2018/B241              AGIGVVGVIAAFIALICICKIIC                                       672
BOMV/M.condylurus/Kenya/2019/X030              AGIGVVGVIAAFIALICICKIIC                                       672
BOMV/M.condylurus/Kenya/2019/Z153              AGIGVVGVIAAFIALICICKIIC                                       672
                                               *****.*** *.***:****::
```

Supplementary Figure S1. Sequence alignment of the EBOV GP sequence to four BOMV isolates GP. Residues involved in direct interaction with the NPC1 receptor are outlined in black, with the BOMV S146 polymorphism highlighted in red. The highly-conserved coiled-coil domain is outlined in blue with sequence polymorphisms at P558 and D607 highlighted in red for the single BOMV isolate from *C. pumilus*

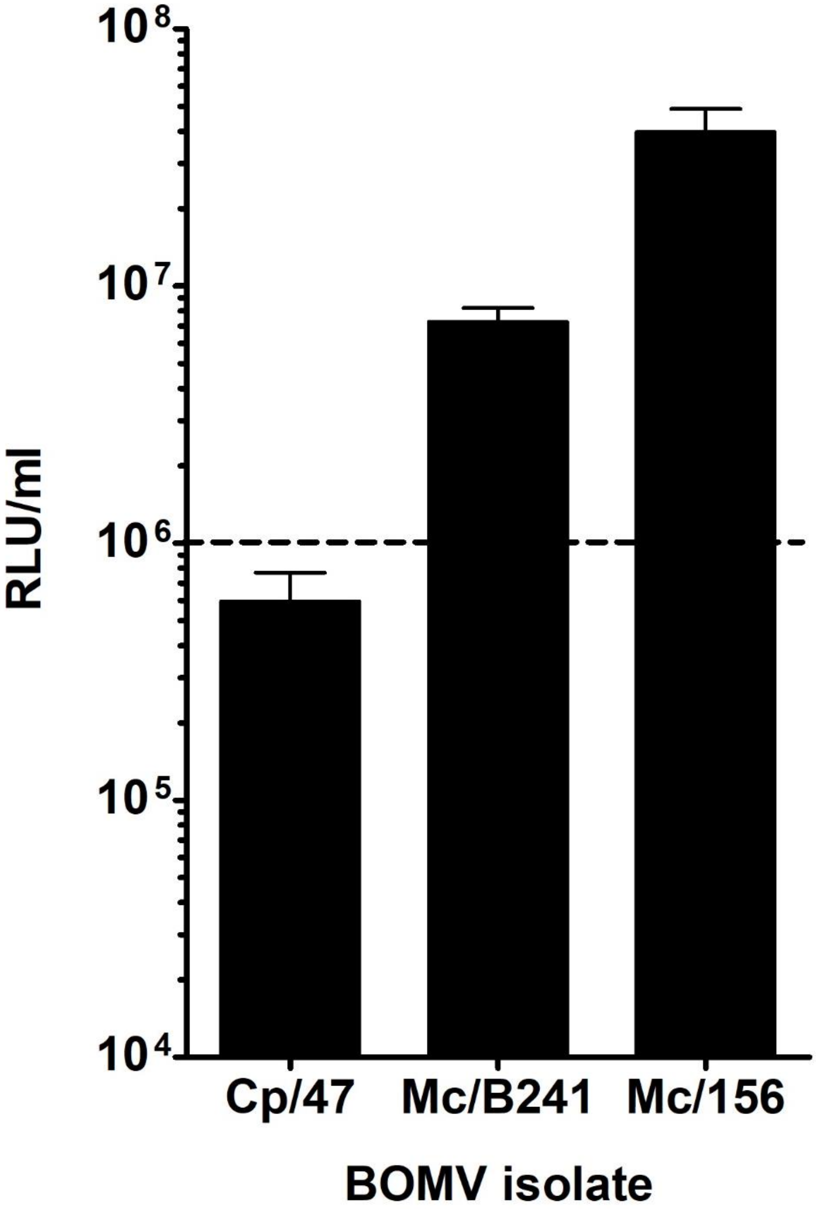

Supplementary Figure S2. Mean titres of lentiviral pseudotype bearing different Bombali ebolavirus (BOMV) isolate GP. Supernatant from LVV producer cells for *C. pu-milus*/SLE/2016/PREDICT_SLAB000047 (Cp/47), *M. condylurus*/Kenya/B241 (Mc/B241) and *M. condylurus*/SLE/2016/PREDICT_SLAB000156 (MC/156) were titrated onto HEK 293T/17 cells. The dotted line indicates the minimum titre required for use in downstream assays. Error bars indicate SEM (n=2).
